# First Observation of *Leptospira interrogans* in the Lungs of *Rattus norvegicus*

**DOI:** 10.1155/2016/9656274

**Published:** 2016-10-05

**Authors:** Anne-Laure Zilber, Patrick Belli, Marc Artois, Angeli Kodjo, Zoheira Djelouadji

**Affiliations:** ^1^USC 1233 INRA/VAS, Université de Lyon-VetAgro Sup, Marcy-l'Etoile, France; ^2^UP Pathologie Morphologique et Clinique, Université de Lyon-VetAgro Sup, Marcy-l'Etoile, France; ^3^Laboratoire des Leptospires, Université de Lyon-VetAgro Sup, Marcy-l'Etoile, France

## Abstract

We report the first two cases of pulmonary presence of leptospires in apparently healthy rats captured in a city park in Lyon (France). Only renal carriage of* Leptospira* has been described in the literature. Blood serology was performed in parallel with molecular and histological analyses of the kidney and lung samples. We isolated leptospires from the kidneys of two out of three seropositive wild rats. These results were confirmed by specific detection of pathogenic* Leptospira* by real-time PCR. Moreover,* Leptospira* DNA was detected in lung tissues. Immunohistochemistry and Warthin-Starry staining revealed that leptospires were present on the surface of the ciliated epithelium of the bronchi. Using PCR of the* rrs* (16S) gene and Multispacer Sequence Typing, DNA extracts of the kidney and lung were identified as belonging to* Leptospira interrogans* serovar Icterohaemorrhagiae “CHU Réunion.” This first observation of the presence* Leptospira* in the lung with simultaneous renal carriage will require further study in future on several target organs to gain a better understanding of the* Leptospira* infection in wild rat.

## 1. Introduction

Leptospirosis is a reemerging zoonosis worldwide [1, 2]; that is caused by a spirochete of genus* Leptospira*. The WHO reports that one million severe human cases of leptospirosis arise each year [2], especially in tropical regions. Metropolitan France has the highest incidence of infection among developed countries [2].

Leptospirosis has several clinical presentations. Weil's disease represents the most frequent severe form in humans, with a fatality rate ranging from 5% to 15%. The clinical manifestations are characterized by jaundice, renal failure, and hemorrhages [1]. Over the last two decades, the incidence of pulmonary involvement in humans [1, 3, 4] and in dogs [5, 6] has increased; thus, it has become a major clinical manifestation of the illness. Several studies have shown the emergence of* Leptospira* strains that cause renal and pulmonary signs in humans and a range of animal species [4, 7], and the pulmonary localization of these strains has been studied in animal models. In acute infections, leptospires have been found to be abundant in the kidney, spleen, and liver. In contrast, few leptospires have been detected in lung tissue, especially in the alveolar septal wall [8].

Most mammalian species are likely to carry leptospires; however, the primary maintenance hosts are wild rodents, such as the brown rat (*Rattus norvegicus*), which is considered to be a reservoir for these bacteria through renal carriage [1]. In rats, leptospirosis is an asymptomatic infection, and leptospires persist, colonizing the proximal renal tubules [1]. The specific associations between some serovars and animal reservoirs have previously been described, and* Rattus* species have been identified as the main reservoir of the Icterohaemorrhagiae serogroup [1]. Thus, the majority of human cases of leptospirosis are caused by the Icterohaemorrhagiae serogroup [2], which is the most frequently observed serogroup worldwide [1, 2]. Consequently, many epidemiological studies have investigated renal carriage in rodents in endemic or nonendemic regions through the assessment of* Leptospira* in both the urine and the kidney; however, few studies have monitored the presence of leptospires in other organs. Moreover, an experimental approach in a rat model revealed the transient passage of leptospires through the lungs and several organs during infection.* Leptospira* was detected in the lung at five days after inoculation, and this was followed by a clearance phase [9]. Thus, hallmarks of the chronic infection model are renal carriage and the absence of leptospires in the lungs.

In the context of an investigation of the microbiota in a few free-living rats (*Rattus norvegicus*) by using several reference detection methods, we evaluated the presence of* Leptospira* in several target organs, which were collected to serve as possible positive controls in future analyses. As a consequence, in the present study, we report two cases in which leptospires were simultaneously present in the kidneys and lungs of rats.

## 2. Materials and Methods

Rats were captured during a routine intervention in our field study area, which has a high level of* Leptospira*'s carriage in rats [10]. In a short-term survey of the microbiota, potentially infected animals were caught. The specimens of free-living Norway rats were identified as* Rattus norvegicus* based on their morphological characteristics [11].

Five traps were set in a city park in Lyon (France) in January 2013 (one week). Trapped rats were transported to the laboratory and were immediately anaesthetized using isoflurane and sacrificed by cervical dislocation, in accordance with the ethical standard of the European Union Legislation governing the care and use of laboratory animals (Directive EU 86/609). Rats were trapped for the purpose of pest control (permit for capture number 69-1810). They were euthanized and used according the ethical rules approved by the Rhône Préfecture (agreement number 69-127811). Data regarding the rats' weight, size, sex, and sexual maturity were recorded. Blood and tissue samples were aseptically collected and treated according to the aims of the serological, histological, and molecular analysis.

Serological tests using the microscopic agglutination test (MAT) [12] were performed in the Laboratoire des Leptospires (VetAgro Sup, Marcy-l'Etoile, France). Blood samples were centrifuged, and the serum obtained was stored at −20°C. A total of 23* Leptospira* strains belonging to 14 serogroups were used for the MAT: Australis (using the Australis, Bratislava, and Munich serovars as antigens), Autumnalis (using the Autumnalis and Bim serovars), Ballum, Bataviae, Canicola, Grippotyphosa (using the Grippotyphosa and Vanderhoedoni serovars as antigens), Hebdomadis, Icterohaemorrhagiae (using the M20 Copenhageni and Verdun Icterohaemorrhagiae serovars), Panama (using the Panama and Mangus serovars as antigens), Pomona (using the Pomona and Mozdok serovars), Pyrogenes, Sejroe (using the Sejroe, Saxkoebing, Hardjo, and Wolffi serovars), Tarassovi, and Cynopteri. The screening was performed starting with a serum dilution of 1 : 30 up to a dilution of 1 : 480. The endpoint was the highest serum dilution showing 50% agglutination in free-moving leptospires. A titer of 1 : 100 or more was considered positive, and this titer was also used in several previous studies [13, 14].

For* Leptospira* isolation, half of one kidney from each rat was crushed and aseptically transferred to tubes containing Ellinghausen McCullough Johnson and Harris (EMJH) medium (Indicia, St Génis, France) [15]. Three serial dilutions tubes were incubated at 29°C according to the protocol for pathogenic* Leptospira* isolation [15]. For a period of three months, the tubes were examined weekly using a dark-field microscope.

For molecular analyses, samples of each organ (kidney, liver, spleen, and lung) were aseptically homogenized using a syringe. After digestion, DNA was extracted from 200 *μ*L of lysed tissue or 200 *μ*L of lysed* Leptospira* culture using the QIAamp DNA Mini Kit (Qiagen, Courtaboeuf, France) following the manufacturer's instructions. All the DNA samples were stored at −20°C. The presence of* Leptospira* DNA was assessed using a specific pathogenic* Leptospira* TaqMan real-time PCR kit (TaqVet PathoLept, LSI, France). As an appropriate negative control, PCR mix without the target DNA was included. The specimens with a Cycle threshold (Ct) of less than 45 cycles were considered positive, in accordance with the manufacturer's instructions.

All positive samples were then characterized in two phases: species identification using the* rrs* (16S) gene [16], followed by genotyping using Multispacer Sequence Typing (MST) [17]. For the first step, DNA was amplified using the* rrs* (16S) gene primers (LeptA, LeptB). The PCRs were performed in a final volume of 50 *μ*L containing 35 *μ*L of H_2_O, 5 *μ*L of 10x buffer (Qiagen), 2 *μ*L of 25 mM MgCl_2_, 1 *μ*L of 10 mM dNTPs (Qiagen), 1 *μ*L of forward primer (10 *μ*M), 1 *μ*L of reverse primer (10 *μ*M), 0.3 *μ*L of HotStarTaq DNA Polymerase (Qiagen), and 5 *μ*L of target DNA. The following thermocycling program was utilized: a 15-min enzyme activation step at 95°C, followed by 40 cycles of 95°C for 30 s, 57°C for 30 s, and 72°C for 1 min with a final elongation step of 72°C for 10 min. PCR mix without the target DNA was included as a negative control, and PCR mix with* Leptospira interrogans* serovar Copenhageni strain Fiocruz DNA was included as a positive control. The PCR products were sequenced with the BigDye Terminator sequencing kit using a 3730XL DNA analyzer (Applied Biosystems, Saint Aubin, France). The* Leptospira* species were identified using the sequence information from NCBI nucleotide BLAST (http://blast.ncbi.nlm.nih.gov/). For the second step, the samples were characterized using MST, as previously described [17]. PCR mix without the target DNA was included as a negative control, and PCR mix with* Leptospira interrogans* serovar Copenhageni strain Fiocruz DNA was included as a positive control. The PCR products were sequenced with the BigDye Terminator sequencing kit using a 3730XL DNA analyzer (Applied Biosystems, Saint Aubin, France). The MST profile of the sequences was determined using NCBI nucleotide BLAST (http://blast.ncbi.nlm.nih.gov/).

For the histological analyses, two stains were performed for visualization of the leptospires; Warthin-Starry silver staining was used as a complement to immunohistochemistry targeting the* L. interrogans* serovar Icterohaemorrhagiae. Samples of each organ (kidney, liver, spleen, and lung) were fixed in 10% formaldehyde for 24 h and subsequently transferred to 70% ethanol. The tissues were embedded in paraffin and cut into 3-*μ*m sections. For each rat, all the organ samples were embedded in the same paraffin block. For each rat, one section was stained with Warthin-Starry silver staining (Merck KGaA, Darmstadt, Germany) [18], and another section was subjected to immunohistochemistry with antiserum specific to the* L. interrogans* Icterohaemorrhagiae serovar. For immunohistochemistry, the paraffin was removed from the sections with xylene and ethanol. The tissues were incubated in a citrate buffer (pH = 6) for 1 h at 95°C and subsequently treated with 0.3% hydrogen peroxide for 10 min at room temperature. The nonspecific sites were blocked by incubation of the sections with Super Block (UltraTek HRP Anti-Polyvalent Lab Pack, ScyTek Laboratories, Logan, USA) for 30 min at room temperature, and the rodent-specific sites were blocked by incubation with a 1,000-fold dilution of peroxidase-conjugated goat anti-rat antibody (Jackson ImmunoResearch Laboratories, West Grove, USA) for 10 min at room temperature. Tissue sections were incubated with a 2,000-fold dilution of* Leptospira* Icterohaemorrhagiae antiserum (Institut Pasteur, Paris, France) overnight at 4°C. The samples were then incubated with a 1 : 2 dilution of UltraTek Anti-Polyvalent (UltraTek HRP Anti-Polyvalent Lab Pack, ScyTek Laboratories) for 30 min at room temperature; subsequently, they were incubated with UltraTek HRP (UltraTek HRP Anti-Polyvalent Lab Pack, ScyTek Laboratories) at room temperature for 30 min. Enzymatic reactions were developed using the Vector NovaRED substrate kit for peroxidase (Vector Laboratories, Burlingame, USA). As an appropriate negative control, sections were included that were incubated without* Leptospira* antiserum.

## 3. Results

During 5 days of trapping, three Norway rats were captured alive. All the rats were male adults, and they were sexually mature, as indicated in Table 1. The general condition of each rat was acceptable, and the rats appeared healthy. In a previously investigated site with a high level of infected animals, only a few animals were needed to constitute a sample of infected rats. The three rats that we captured served as controls for further tests. The rats were named DTO14, DTO15, and DTO16.

Serological tests showed that the three rats were positive using the MAT, and all had antibody titers of 1 : 120 for the Icterohaemorrhagiae serovar and 1 : 30–1 : 60 for the Copenhageni serovar (Table 1). These two serovars belong to the Icterohaemorrhagiae serogroup. Moreover, DTO14 had antibody titers of 1 : 120 for the Mangus serovar (Panama serogroup) and the Cynopteri serovar (Cynopteri serogroup). DTO15 had an antibody titer of 1 : 120 for the Cynopteri serovar.

*Leptospira* isolation showed that only two of the three rats (DTO14 and DTO16) produced positive renal cultures, as shown in Table 1.

Molecular analyses were performed on DNA extracts from the target organs of the three rats. The results showed that DTO14 tested positive in the kidney and the lung (20 and 34 Ct, resp.). PCR indicated that the kidney and lung from DTO15 were negative for pathogenic* Leptospira.* Finally, for DTO16, PCR of the DNA extracts from the kidney (24 Ct) was positive, whereas that from DNA extracts of the lung was negative, even after repeated runs. All the results are shown in Table 1. The positive control yielded 25 Ct on average. All liver and spleen samples from the three rats had negative qPCR results.

Using* rrs* (16S) gene amplification, the leptospires detected from the lung, kidney, and corresponding isolates were identified as belonging to the species* Leptospira interrogans*. Further, MST genotyping showed that the sequences of all the samples were identical to the sequences of the Icterohaemorrhagiae serogroup, Icterohaemorrhagiae serovar “CHU Réunion” profile, with a strict similarity between leptospires from the lung and from the kidney.

**Figure 1:**
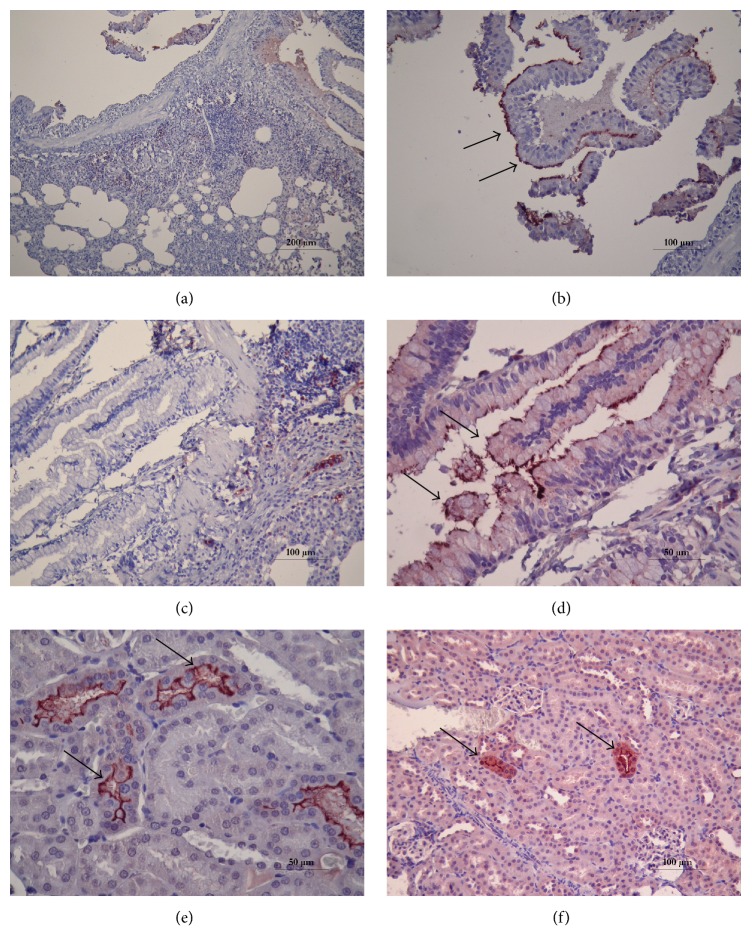
Immunohistochemical examination of the kidney and lungs of wild rats naturally infected with* Leptospira*. (a, c) Pulmonary sections obtained from two rats, DTO14 and DTO16, which were not incubated with anti-leptospire antibodies (negative control; no stain fixation). (b, d) Pulmonary sections obtained from the same rats showing the presence of leptospires (arrows) on the bronchial epithelium using anti-leptospire antibodies. (e, f) Renal sections obtained from two rats, DTO14 and DTO16, showing dense* Leptospira* colonization in the renal tubules using anti-leptospire antibodies (arrows).

Immunohistochemical staining of the tissue sections of DTO14 and DTO16 using specific anti-leptospire antibodies revealed that leptospires were localized on the surface of the bronchial ciliated epithelium, as shown in Figures 1(b) and 1(d); these results were validated by negative controls in which staining was performed without anti-leptospire antibodies (Figures 1(a) and 1(c)). In the same rats, leptospire colonization was observable in several tubules, as shown in Figures 1(e) and 1(f). Renal and pulmonary sections from the third rat, DTO15, were negative. All liver and spleen samples from the three rats had negative results with immunohistochemistry and the Warthin-Starry staining.

**Figure 2:**
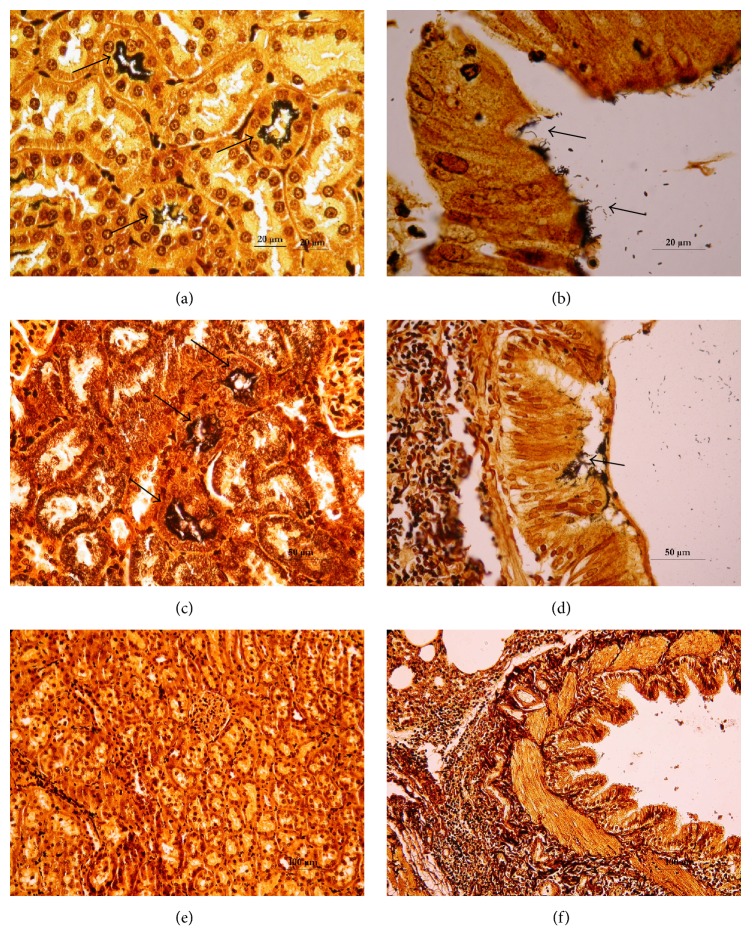
Histopathological examination of the kidney and lungs of wild rats naturally infected with* Leptospira* using Warthin-Starry silver stain. (a, c) Renal sections obtained from two rats, DTO14 and DTO16, showing dense* Leptospira* colonization in the renal tubules (approximately 3 *μ*m thick) (arrows). (b, d) Pulmonary sections from the same rats showing the presence of leptospires (arrows) on the bronchial epithelium. (e, f) Renal and pulmonary sections of the third rat, which was negative.

The silver-stained sections also revealed that DTO14 and DTO16 had very dense leptospire colonization in their renal tubules (Figures 2(a) and 2(c)). However, the number of colonized tubules relative to the total number of viewed tubules was low, and their distribution was heterogeneous. The renal structures were histologically normal, and no sign of an immune system reaction was observed around the infected tubules. In the same rats (DTO14 and DTO16), silver-stained leptospires were also detected on the bronchial epithelium, as shown in Figures 2(b) and 2(d). Leptospires were observed in the bronchi but not in the alveolar sacs. They localized at the level of the ciliated epithelium, with a heterogeneous and compact distribution. Renal and pulmonary sections obtained from the third rat, DTO15, were negative (Figures 2(e) and 2(f)). In addition, we observed pulmonary lymph node hypertrophy in the lung sections of all rats. Therefore, in DTO14 and DTO16, leptospire localization was identical as observed by both immunohistochemistry and silver staining. In these rats, leptospires were localized on the surface of the bronchial ciliated epithelium and the renal tubules.

## 4. Discussion

In the present study, we report the simultaneous presence of leptospires in the renal tubules and on the bronchial epithelium in two free-living rats (*Rattus norvegicus*) for the first time.

The context of our investigation precluded intentionally the evaluation of the prevalence of* Leptospira*. In order to collect infected tissues for use as positive controls in later studies, these tissues were originally collected from just a few captured animals in an area in which the wild rats had previously been characterized as having a high prevalence of leptospirosis [10].

All three trapped rats (DTO14, DTO15, and DTO16) were seropositive. The low antibody titer might suggest that these rats have recovered from infection. However, in DTO14 and DTO16, leptospires were detected in both the lung and the kidney with molecular and histological methods, and these findings were confirmed by renal isolation. Thus, a low antibody titer might result from adaptation between the host and the pathogen [19], which was characterized by the presence of the bacteria in the renal tubules, an immune escape site [20]. Furthermore, despite very dense colonization of the leptospires, as shown by silver staining, lesions were not observed in the renal tissues. An inflammatory reaction did not appear to be correlated with the isolation of leptospires or the presence of leptospires [20, 21]. Thus, the absence of interstitial nephritis was possible, even if this latter was the only morphological alteration attributable to leptospiral infection [22]. Therefore, the rats DTO14 and DTO16 could be considered chronically infected based on the dense renal colonization by* Leptospira*.

To the best of our knowledge, this is the first time that leptospires have been found in the lung tissues of free-living rats. Their presence was detected via a molecular method and confirmed by histological analysis. Leptospires were observed by immunohistochemistry (heterogeneous and compact distribution of leptospires on the ciliated epithelium) with Warthin-Starry staining used as a complement. However, pulmonary lymph node hypertrophy was observed in both infected and noninfected rats and could be caused by an immune response against an incidental contaminant. Furthermore, genotyping showed that the leptospires present in both the bronchi and the renal tubules belonged to the Icterohaemorrhagiae serogroup, Icterohaemorrhagiae serovar “CHU Réunion” strain of* Leptospira interrogans*. Thus, the renal and pulmonary leptospires could have originated from the same infectious source. It is necessary to investigate the origin of this pulmonary localization by other studies to identify the route of* Leptospira*'s contamination (via air-borne or blood-borne route).

Several techniques were utilized in our study. The isolation of the leptospires from pulmonary tissues was not feasible because only two small pieces of lung were available for molecular and histological analyses. The presence of leptospires on the surface of the bronchial ciliated epithelium was validated by three control points. The first was simultaneous staining of the renal and pulmonary leptospires using the same paraffin block containing a piece of the kidney and a piece of the lung. The second was the use of an optimized immunohistological protocol that involved the addition of specific anti-rat antibodies and the use of specific* Leptospira* antibodies targeted against the Icterohaemorrhagiae serogroup. The third was a negative immunohistochemical control involving staining without the use of anti-leptospire antibodies. The heterogeneous and compact distribution of the leptospires on the ciliated epithelium cannot be confused with other anatomical features of the bronchi. The presence of leptospire DNA in the lung was verified by several molecular methods: a specific pathogenic* Leptospira* real-time PCR protocol used in a previous survey of wild rats [10], PCR of the* rrs* (16S) gene, and typing with an MST protocol previously tested on rats' renal isolates [17]. The negative PCR result obtained with the lung tissues of DTO16 could be explained by a sampling bias that may have occurred during sample collection for DNA extraction, as leptospires were located in small clusters in some bronchi. A similar failure of detection was previously reported in infected kidney tissue (Warthin-Starry-positive kidney with negative PCR results and a negative culture) [21].

This study demonstrated the simultaneous presence of leptospires in the renal tubules and the bronchi in wild rats. This observation of natural infection was different from previous observations of experimental infection in rats, in which* Leptospira* was not detected (or rarely) in the lung during the infection (in both the acute and chronic phases) [9, 23]. Moreover, in contrast to what was observed during acute infection, the leptospires were localized on the ciliated epithelium of the bronchi and not in the alveolar sacs of experimentally infected guinea pigs [8].

The observation of the pulmonary presence of* Leptospira* in these two cases raises questions about the origins of this location and the hypothesis of potential pulmonary presentation of* Leptospira* infections in wild rats. Future epidemiological studies should involve examinations of the presence of leptospires in rat's lung using histological and molecular methods to gain a better understanding of the* Leptospira* infection in rats.

## 5. Conclusions

For the first time, we report the simultaneous presence of leptospires in the renal tubules and on the bronchial epithelium in two free-living rats (*Rattus norvegicus*), in an area in which the wild rats had a high prevalence of leptospirosis. Future epidemiological studies should involve molecular and histological examinations of the lung to answer questions about the origins of this rare location, which could allow us to better understand the evolutionary association between the rat and* Leptospira*.

## Figures and Tables

**Table 1:** Results of various laboratory tests performed on three *Rattus norvegicus* individuals naturally infected in a city park in Lyon (France).

Rat	Weight (g)	Size (mm)	Sex	Age	MAT (serovar)	Organs	*Leptospira* isolation	Real-time PCR (Ct)	Immunohistochemistry	Warthin-Starry staining
DTO14	206	235	Male	Adult	Copenhageni: 1 : 60; IH; Cynopteri; Mangus: 1 : 120	Kidney Lung	+ n/a	+ (19, 88) + (34, 15)	+^1^ +^2^	+^1^ +^2^
DTO15	239	243	Male	Adult	Copenhageni: 1 : 60; IH; Cynopteri: 1 : 120	Kidney Lung	− n/a	− −	− −	− −
DTO16	176	225	Male	Adult	Copenhageni: 1 : 30; IH: 1 : 120	Kidney Lung	+ n/a	+ (23, 95) −	+^1^ +^2^	+^1^ +^2^

IH: Icterohaemorrhagiae serovar; +: presence of leptospires or *Leptospira* DNA; −: absence of leptospires or *Leptospira* DNA.

^1^The leptospires visualized on the epithelium of the renal tubules.

^2^The leptospires visualized on the ciliated epithelium of the bronchi.
